# A Retrospective Cross-Sectional Study of Clinical Profile of HIV Patients at a Tertiary Care Hospital in Western Part of Maharashtra

**DOI:** 10.7759/cureus.71954

**Published:** 2024-10-20

**Authors:** Tejas Gadodia, Marcia Waran, Akhilendra B Khare, Arun Tyagi, Awani K Shrivastava, Roshan S Prasad

**Affiliations:** 1 Medicine, Dr. Vithalrao Vikhe Patil Foundation's Medical College and Hospital, Ahmednagar, IND; 2 Medicine, Datta Meghe Institute of Higher Education and Research, Wardha, IND; 3 Community and Family Medicine, Datta Meghe Institute of Higher Education and Research, Wardha, IND

**Keywords:** antiretroviral drugs, cd4 count, hiv, naco, opportunist infections

## Abstract

Background and aim

In India, socioeconomic factors, healthcare access, and cultural views influence HIV epidemiology. With over two million cases, high-risk groups include drivers, sex workers, and intravenous drug users. Early symptoms often go unnoticed, and advanced stages lead to opportunistic infections. Despite antiretroviral therapy (ART) advancements, access remains a challenge. Effective management per National AIDS Control Organization (NACO) guidelines is essential. This study aimed to understand the clinical profile of people living with HIV (PLWH) with sociodemographic variation at tertiary care centers in the western part of Maharashtra.

Methods and methodology

A retrospective descriptive cross-sectional study was conducted on PLWH from November 2022 to May 2024. The PLWH patients of all age groups and all categories were included in the study. The sociodemographic data were collected, and clinical staging was done according to the World Health Organization (WHO) classification of HIV/AIDS. Data were expressed as mean±standard deviation and percentage proportion.

Results

Out of 150 PLWH, 77 (51.33%) were male and 73 (48.67%) were female patients. The majority of patients, 102 out of 150, were in the age group of 30-50 years. The most common mode of HIV transmission was heterosexual (143 patients, 95.33%). At the time of presentation, 102 patients (68%) were symptomatic, while 48 patients (32%) were asymptomatic. A total of 77 patients (51.33%) were included in WHO clinical stage 2 of HIV disease. In terms of cluster of differentiation 4 (CD 4) count, 62 patients (41.33%) had counts between 201 and 500, 54 patients (36%) had counts greater than 500, and 34 patients (22.67%) had counts below 200. Most patients, 94 (62.67%), had a BMI of less than 18.5. Pulmonary tuberculosis was found to be the most commonly associated opportunistic infection (OI), constituting 57 (38%) cases, followed by acute gastroenteritis 35 (23.33%).

Conclusion

The demographic and clinical profiles of the study participants highlight the importance of early case detection and the timely initiation of highly active antiretroviral therapy.

## Introduction

Human immunodeficiency virus (HIV) primarily attacks the immune system by targeting cluster of differentiation 4 (CD4) cells, and if not diagnosed early, it can progress to acquired immunodeficiency syndrome (AIDS), which is a severe immune deficiency [1]. Socioeconomic factors, healthcare access, and cultural views on sexual health influence the epidemiology of HIV in India. Recent data indicates that over two million people in India are living with HIV, with a higher prevalence among high-risk groups like drivers, sex workers, and intravenous drug users [2]. Notably, India ranks third globally in terms of people living with HIV, with an estimated 2.1 million cases in 2019 [3]. There is an uneven distribution of cases of HIV. For instance, states like Tamil Nadu and Andhra Pradesh reported more cases as compared to northern states like Uttar Pradesh and Bihar [4].

Clinical presentation of HIV varies widely and is influenced by the infection stage, co-existing conditions, and individual immune responses. Early HIV symptoms may resemble mild flu-like conditions, such as fever, sore throat, and fatigue, often going unnoticed. In advanced HIV disease with a weakened immune system, patients are susceptible to opportunistic infections [5]. Effective understanding of these clinical manifestations is essential for timely diagnosis and intervention. Despite the advancements in antiretroviral therapy (ART), many patients in India still face challenges in accessing treatment and care [6]. Therefore, the clinical profile of HIV patients in India is complex and requires a comprehensive approach to manage and improve health outcomes in accordance with National AIDS Control Organization (NACO) guidelines.

## Materials and methods

Study setting and design

This retrospective cross-sectional study was conducted at Dr. Vithalrao Vikhe Patil Foundation's Medical College, Ahmednagar, India, after obtaining ethical clearance from the Institutional Ethical Committee (#VIMS/IEC/C/2022/34), along with written informed consent from the patients. The study was conducted at a tertiary care center in Ahmednagar. The Strengthening the Reporting of Observational Studies in Epidemiology (STROBE) guidelines were followed when conducting the study. The hospital records of the study period were reviewed. Incomplete records and patients who were shifted outside of our makeshift hospital before completing the treatment were excluded from the analysis.

The research spanned over one and a half years, from November 2022 to May 2024, during which 5064 patients attended our center and were screened for HIV infection. Out of these, 150 individuals tested positive for HIV and were included in the study to observe the pattern of opportunistic infections (OIs). Detailed interviews and socioeconomic data were collected from these patients using a structured questionnaire (tables in appendix). Newly diagnosed patients were referred to the district ART center for CD4 count estimation, and for those already on antiretroviral therapy (ART), their recent CD4 counts were considered. At the ART center, free ART medications were provided based on CD4 counts. These patients were monitored at our hospital for the initiation of therapy and any complications that arose.

Selection criteria

A total of 150 patients were enrolled. The inclusion criteria consist of patients admitted to the medicine ward or medicine intensive care unit (ICU) with a confirmed diagnosis of HIV/AIDS, as well as people living with HIV referred from other departments between November 2022 and May 2024. The exclusion criteria involve pediatric patients up to 15 years of age, immunocompromised individuals who have undergone organ transplants, patients on immunosuppressive or steroidal therapy, or those with a known diagnosis of diabetes mellitus.

Data sources and variables

A total of 150 patients enrolled in the study were categorized into three groups according to CD4 count based on the Centers for Disease Control and Prevention (CDC) guidelines. CD4 counts >500 cells/μL included in group 1, group 2 covers counts between 200 and 499 cells/μL, and <200 cells/μL included in group 3 [7]. Although the patient's viral load is crucial for starting and monitoring antiretroviral therapy (ART), our study did not include viral load estimation due to its high cost. According to the protocol of NACO, all patients received ART. Triple drug combination is the most commonly prescribed regimen based on nucleoside reverse transcriptase inhibitors (NRTIs), specifically tenofovir, lamivudine, and dolutegravir (integrase inhibitors). Opportunistic infections (OIs) for each group were documented. Various investigations were done based on clinical symptoms and samples such as sputum, throat swabs, blood, stool, urine, chest X-ray, high-resolution computed tomography (HRCT) thorax, cerebrospinal fluid (CSF), and lymph node aspirates were collected using universal aseptic precautions in appropriate sterile containers. The OIs among these patients were managed according to NACO guidelines at our hospital.

Statistical analysis

Statistical data was compiled into a master graphic using Microsoft Excel (Redmond, WA: Microsoft Corp.) and analyzed with SPSS version 20 (Armonk, NY: IBM Corp.). Descriptive statistics summarized the categorical variables in frequencies (n) and percentages (%).

## Results

Table 1 shows out of 150 people living with HIV, 77 (51%) were male and 73 (49%) were female. Most (101, 67.6%) of the patients were in the age group of 30-50 years. The heterosexual (143 patients, 95.3%) mode of transmission of HIV was the commonest mode, followed by hematogenous transmission (two patients, 1.33%). Five patients (3.33%) contracted the virus from unspecified sources. In cases of heterogeneous sexual transmission involving multiple partners, commercial sex workers pose a significant risk. While intravenous drug use is another key risk factor for HIV transmission, our study found that no participants contracted HIV through IV drug use, based on our history collected.

**Table 1:** Demographic profile of patients with HIV/AIDS.

Variables (n=150)	Number (%)
Sex	
Male	77 (51.33)
Female	73 (48.67)
Age group (years)	
18-30	25 (16.67)
31-40	47 (31.33)
41-50	54 (36.00)
>50	24 (16.00)
Residence	
Urban	36 (24.00)
Rural	114 (76.00)
Mode of transmission	
Heterosexual	143 (95.33)
Homosexual	2 (1.33)
Not specified	5 (3.33)
Blood transfusion	0 (0.00)
Marital status	
Married	110 (73.33)
Unmarried	27 (18.00)
Widowed/divorced/separated	13 (8.67)
HIV status of spouse (only 110 were married)	
Positive	72 (65.45)
Negative	38 (34.54)

The most affected age group was 41-50 years, with 54 patients (36.33%), followed closely by the 31-40 years age group with 31 patients (31.33%). Among the patients, 110 (73.4%) were married, and of those, 72 (64.4%) had spouses who were also found to be HIV positive. A total of 114 patients (76%) were from rural areas, whereas 36 patients (24%) were from urban areas.

Table 2 shows that among the 150 patients who presented to the hospital, 102 (68%) were symptomatic, while 48 (32%) were asymptomatic. The most common symptoms reported were fever, weight loss, cough, anorexia, diarrhea, and generalized weakness. A total of 77 patients (51.33%) were included in WHO clinical stage 2, followed by 46 patients (30.67%) who were included in WHO clinical stage 1.

**Table 2:** Clinical profile of patients with HIV/AIDS. TLD: tenofovir (TDF) + lamivudine (3TC) + dolutegravir (DTG); ZLN: zidovudine (AZT) + lamivudine (3TC) + efavirenz (EFV); TLE: tenofovir (TDF) + lamivudine (3TC) + efavirenz (EFV); TLN = tenofovir (TDF) + lamivudine (3TC) + nevirapine (NVP); CD4: cluster of differentiation 4

Variables	Number (%)
Disease status (HIV serology - status)	
Asymptomatic	48 (32.00)
Symptomatic	102 (68.00)
CD4 count	
<200	34 (22.67)
201-500	62 (41.33)
>500	54 (36.00)
BMI (kg/m^2^)	
<18.50 (underweight)	94 (62.67)
18.50-24.99 (normal range)	51 (34.00)
>25.00 (overweight/obese)	5 (3.33)
WHO clinical stage	
Stage 1	46 (30.67)
Stage 2	77 (51.33)
Stage 3	17 (11.33)
Stage 4	10 (6.67)
ART adherence	
Regular	118 (78.66)
Intermittent	32 (21.33)
Regimens	
TLD (TDF+3TC+DTG)	66 (44.00)
ZLN (AZT+3TC+NVP)	31 (20.67)
TLE (TDF+3TC+EFV)	7 (4.67)
TLN (TDF+3TC+NVP)	3 (2.00)
Other regimens	43 (28.8)
ART adherence	
Regular	118 (78.66)
Intermittent	32 (21.33)

In terms of CD4 count, 62 patients (41.33%) had counts between 201 cells/mm^3^ and 500 cells/mm^3^, 54 patients (36%) had counts greater than 500 cells/mm^3^, and 32 patients (22.67%) had counts below 200 cells/mm^3^. The majority of patients, 94 (62.6%), had a BMI of less than 18.5, indicating underweight status. Additionally, 51 patients (34%) had a BMI ranging from 18.5 to 24.9 (normal weight), and five patients (3.33%) had a BMI over 25 (obese).

All patients were on antiretroviral drugs (ART), and newly diagnosed were started on ART according to NACO guidelines. Table 3 shows out of 150 patients, 66 (44%) were on a tenofovir + lamivudine + dolutegravir (TLD) regimen, 31 (20.6%) were on a zidovudine + lamivudine + efavirenz (ZLN) regimen, seven (4.6%) were on a tenofovir + lamivudine + efavirenz (TLE) regimen, and the rest were on other regimens that mostly included ATY/r + NRTIs (atazanavir/ritonavir) and LPV/r + NRTIs (lopinavir/ritonavir). Among these, 118 (78.66%) were regularly adherent to treatment, and the rest, 32 (21.3%), were taking intermittent or stopped ART.

**Table 3:** Spectrum of opportunistic infections in HIV patients. *One patient having more than one opportunistic infection. PCP: *Pneumocystis jirovecii* pneumonia; CD4: cluster of differentiation 4

Opportunistic infections	Number (%)* (total n=150)	Mean CD4 count
Present (n=94, 62.6%)		
Tuberculosis	57 (38.00)	356 (64-873)
Acute gastroenteritis	35 (23.33)	232 (120-650)
Oral/oesophageal candidiasis	31 (20.67)	187 (40-377)
Skin infections	27 (18.00)	143 (36-294)
CNS infections	7 (4.66)	87 (46-178)
Malignancies	6 (4.00)	76 (21-125)
Toxoplasmosis	5 (3.33)	58 (12-82)
PCP	3 (2.00)	36 (5-67)

During this course of hospitalization, patients were also reported to have secondary opportunistic infections. A total of 92 patients (62.6%) suffered from various opportunistic infections. Pulmonary tuberculosis was found to be the most common associated OI, constituting 57 (38%) of the cases, followed by acute gastroenteritis (35, 23.3%). Out of these, diarrhea was due to a mixed infection of *Escherichia coli* and *Cryptosporidium parvum*. Oral and oesophageal candidiasis account for 31 cases (20.6%), out of which five patients suffered from oesophageal candidiasis. A total of 27 patients (18%) suffered from skin infections, of which herpes zoster infection was present in the majority of patients.

CNS infections are present in seven (4.6%) patients, including two patients with encephalitis and five with meningitis. Tuberculosis (TB) meningitis was present in three patients, while cryptococcal meningitis was present in two patients. Six (4%) patients presented with HIV-associated malignancies, which comprised gastric lymphoma in three patients, breast carcinoma in two patients, and anal carcinoma in one patient. Toxoplasmosis and *Pneumocystis jirovecii* pneumonia (PCP) accounted for five (3.3%) and three (2%) patients, respectively.

## Discussion

In our research on the demographic profile of people living with HIV (PLWH), we found that the average age of patients presented with HIV was 37±12 years, and there was slight male predominance at 51%. This aligns with findings from Gajra Raja Medical College in Gwalior, where the average age was 35±11.5 years, and Sardar Vallabh Bhai Patel Hospital in Meerut, where the mean age for males was 34.62±10.2 years and 32.12±9.62 years for females, with males comprising 66.75% of the sample [8,9]. Similarly, a study from Sawai Man Singh (SMS) Hospital in Jaipur reported an average age of 36±13 years, with males representing 59.16% of the study population [10]. Additionally, we found that 46.66% of participants were within the reproductive age group, compared to 78% in a study conducted at the Institute of Medical Sciences, Banaras Hindu University in Varanasi, and 51% at SMS Hospital in Jaipur [11].

The primary cause of HIV transmission in our study was heterosexual contact, consistent with numerous previous studies on HIV patients' sociodemographic characteristics; however, IV drug users' blood transfusion and vertical transmission were other modes of spread. Studies indicate that female partners are more susceptible to HIV infection than male partners. This trend was also observed in research conducted at a rural tertiary care hospital in Maharashtra, where HIV positivity rates were higher among women. This increased vulnerability in women could be due to the higher concentration of HIV in semen compared to vaginal or cervical fluids and the larger exposed surface area [12].

In our study, 76% of patients resided in rural areas, with only 24% living in urban areas. Joge et al.'s study also suggests similar findings [12]. Illiteracy and lack of awareness regarding safe sex practices might contribute to the high prevalence of HIV in rural areas. Most participants in our study were married. Various factors can increase the risk of HIV infection in marriage, especially for women, including marginalization, disempowerment, and geographical barriers that limit access to HIV knowledge and services.

Diagnoses in our study were primarily made based on clinical symptoms, but 32% of patients who were diagnosed to be HIV positive were asymptomatic at presentation. The majority of patients were classified under WHO clinical stage 2 (51.33%) and stage 1 (30.6%), indicating that they were in relatively intact immune health and early stages of the disease. These findings correlate with studies conducted at SMS Jaipur and Lok Nayak Hospital in New Delhi. However, 42.5% of patients were in stage 3 in a study conducted at a rural tertiary care hospital in Maharashtra, which does not correlate to our study [13].

Our sample revealed that 58.6% of HIV patients had a BMI <18.5 (underweight). Consistent with previous research, patients who were non-adherent with ART were found to have lower BMI and a higher risk of AIDS-associated opportunistic infections. CD4 count remains a reliable measure for clinical staging and treatment decisions, alongside identifying opportunistic infections (OIs). The prolonged non-adherence to antiretroviral drugs is marked by a decline in CD4+ T helper cells and persistent viral replication, leading to immunologic decline and death from OIs and neoplasms. Most of the patients reported in critical condition attributed to multiple opportunistic infections and decreasing CD4 counts.

In this study, the combinations of TLD and ZLN were prescribed to 44% and 20.66% of patients, respectively. Although ART does not cure HIV/AIDS, effective regimens inhibit viral replication and reduce viremia. Lifelong treatment adherence is crucial and involves consistent HIV medication intake, regular medical appointments, and following prescriptions accurately. In comparison, the study by Deshpande et al. reported that ZLN was prescribed to 47.3% and SLN was prescribed to 31.5% of patients, respectively [14]. Our study showed that the majority of patients (78.66%) are compliant with ART, reflecting the effectiveness of ART centers. Poor ART compliance is associated with less effective treatment, risking patient health, and potential treatment resistance to specific agents within combination therapy regimens.

Most patients presented with chief complaints of fever, cough, anorexia, and weight loss, which were comparable to other studies. The range of opportunistic infections in our study aligns with those from past research at Gajra Raja Medical College, Gwalior [8]. However, we observed higher rates of candidiasis and other infections. Tuberculosis remains the most common opportunistic infection, followed by acute gastroenteritis and candidiasis, similar to other studies in India. In some cases, candidiasis ranks just behind tuberculosis as the leading opportunistic infection. In our country, where tuberculosis is widespread, HIV compromises the immune system, making tuberculosis more active. We also noted skin infections like herpes zoster, dermatitis, warts, and cellulitis, with herpes zoster being the most prevalent. Additionally, conditions like encephalitis, meningitis, toxoplasmosis, and HIV-related cancers were observed, consistent with other research findings [9-11].

There is an elevated number of late-stage HIV diagnoses, indicating delayed or inaccessible healthcare. The high prevalence of TB coinfection among HIV patients suggests the need for integrated care models [15]. There were varied adherence levels to antiretroviral therapy (ART) across different regions, potentially due to access or cultural factors. Significant gender disparities in HIV care were observed, with women facing more barriers to treatment and support services. Trends show an increasing rate of HIV among youth, signaling the importance of targeted prevention strategies for this demographic pattern.

Limitations

This study is a retrospective cross-sectional analysis based on documents, which may contain missing information. The treatment regimens were inconsistent since patients entered the study at different times. Investigations such as viral load were not included due to financial constraints. Other factors, like income status and education level, were not considered because it is a retrospective study. Opportunistic infections were categorized broadly because detailed studies of the causative organisms were not possible due to the lack of specific tests at this center. The analysis was conducted at a single center and relied on historical records and self-reported behavior. To gain a better understanding of the adolescent HIV epidemic in India, multicentric and prospective studies with larger sample sizes are needed. This study, limited to Western Maharashtra, does not represent the entire country.

## Conclusions

Our study found that a significant number of patients were from rural areas, predominantly within the reproductive age group, with a higher prevalence of males and a BMI of less than 18.5. Upon their initial hospital visit, most patients were asymptomatic and classified within WHO clinical stages 1 and 2. The primary reason for hospital visits was opportunistic infections, which are the leading cause of morbidity and mortality among HIV patients. Pulmonary tuberculosis was the most common opportunistic infection, followed by gastroenteritis and candidiasis. Therefore, early diagnosis and the initiation of highly active antiretroviral therapy (HAART) are crucial.

Implications of our study suggest a need for more targeted public health interventions that address the specific needs of HIV patients in India, such as tailored prevention strategies and improved access to care. Future research could focus on understanding the socioeconomic and cultural factors that contribute to the high prevalence of HIV in certain regions of India in order to develop more effective community-based interventions. These findings could inform the development of national health policies that prioritize HIV prevention and care, ensuring that resources are allocated to areas with the greatest need. The research also highlights the importance of ongoing surveillance and monitoring of HIV trends in India to track progress and identify emerging challenges in the fight against HIV/AIDS.

## Appendices

**Table 4:** Questionnaire proforma for data collection and analysis.

Characteristics		Responses
Name		
Age (years)		
Gender	Male	
	Female	
Occupation		
Presenting complaints		
Past history		
Previous blood transfusions	Yes	
	No	
Marital status	Married	
	Unmarried	
Spouse HIV status	Yes	
	No	

**Table 5:** Clinical examination protocols for data collection.

Clinical investigations	Values
General examination	
Consciousness	
Pallor	
Jaundice	
Lymphadenopathy	
Vitals	
Pulse rate	
Blood pressure	
Respiratory rate	
SpO_2_	
Systemic examination	
Cardiovascular	
Respiratory	
Per abdomen	
Central nervous system	

**Table 6:** Questionnaire for laboratory investigations.

Laboratory tests	Values
Complete blood count (CBC) Hb/Total leukocyte count (TLC)/platelets	
Chest X-ray (CXR)	
Electrocardiograph (ECG)	
CD4 count	
Cerebrospinal fluid (CSF) analysis	
Throat swab	
Blood culture	
Viral load	
Liver function test (LFT)	
Kidney function test (KFT)	
Fasting and post-prandial blood glucose	
Urine routine	
High-resolution computed tomography (HRCT) chest	
Thyroid function tests (TFT)	
Lymph node aspirate	
Troponin I	
HbA1c	
